# mSphere of Influence: Intermetal Competition during Manganese Toxicity

**DOI:** 10.1128/mSphere.00949-19

**Published:** 2020-01-08

**Authors:** Julia E. Martin

**Affiliations:** a Department of Biological Sciences, Idaho State University, Pocatello, Idaho, USA

**Keywords:** *Streptococcus*, metal homeostasis, manganese

## Abstract

Julia E. Martin works in the field of metals in biology, with a focus on manganese (Mn) homeostasis in Streptococcus pneumoniae. In this mSphere of Influence article, she reflects on how the paper entitled “Role of the manganese efflux system *mntE* for signalling and pathogenesis in Streptococcus pneumoniae” (J. W. Rosch, G. Gao, G. Ridout, Y.-D. Wang, and E. I. Tuomanen, Mol Microbiol 72:12–25, 2009, https://doi.org/10.1111/j.1365-2958.2009.06638.x) has impacted her thinking and research direction toward investigating the molecular underpinnings of why and how bacteria maintain optimal intracellular Mn levels.

## Abstract (precis)

Julia E. Martin works in the field of metals in biology, with a focus on manganese (Mn) homeostasis in Streptococcus pneumoniae. In this mSphere of Influence article, she reflects on how the paper entitled “Role of the manganese efflux system *mntE* for signalling and pathogenesis in Streptococcus pneumoniae” (J. W. Rosch, G. Gao, G. Ridout, Y.-D. Wang, and E. I.

## COMMENTARY

An important aspect of bacterial survival is the ability to sense and respond rapidly to various stress conditions and environmental changes, including transition metal ion fluctuations. Transition metals from manganese (Mn) to zinc (Zn) serve as cofactors in enzymes involved in many critical biological processes. Bacteria must acquire these essential metal ions exogenously in sufficient quantity to fulfill their cellular demand. This metal essentiality is exploited by the mammalian host immune system through nutritional immunity, in which transition metal ions are sequestered by the host in an effort to limit growth of invading bacteria. Historically, many studies have focused on metal uptake and other mechanisms of bacterial adaptation to host-imposed metal starvation. This is particularly true of Mn, an important micronutrient that is largely recognized for its role in oxidative stress resistance and in enhancing virulence of bacterial pathogens. Although elusive, it was revealed almost half a century ago that excess Mn can impair bacterial growth (Fisher et al., 1973 [1]; Silver et al., 1972 [2]). Today, we find that researchers have identified several different transport systems in bacteria that are capable of exporting Mn. The article “Role of the manganese efflux system *mntE* for signaling and pathogenesis in Streptococcus pneumoniae” is among the first to identify one type of putative Mn exporter and to demonstrate that Mn efflux is necessary for host pathogenesis (Rosch et al., 2009 [3]). That article also revealed possible roles for intracellular Mn beyond activating superoxide dismutase in bacteria.

Briefly, on the basis of sequence homology, the authors of that article identified a conserved gene sequence, S. pneumoniae 1552 (SP_1552; designated *mntE*), encoding a cation diffusion transporter. Mn became the prime suspected substrate after metal cation sensitivity profiles and intracellular metal composition measurements obtained by inductively coupled plasma-mass spectrometry of null-*mntE* mutants showed reduced growth and elevated Mn concentrations, respectively, compared to unstressed wild-type cells. Mn isotopic uptake assays further demonstrated that the *mntE* mutant deficient in metal export accumulates significantly higher levels of intracellular Mn, consistent with the hypothesis that MntE functions in Mn efflux. A role for Mn efflux in host pathogenesis was observed by assessing the virulence of the *mntE* mutant in the intranasal murine mouse model of infection. Given that the *mntE* mutant was attenuated, Rosch et al. analyzed the global transcriptome of pneumococcus grown under various conditions of Mn stress. The transcript profiles obtained were used as a mechanism to elicit which metabolic pathways might be important for pneumococcus in establishing disease. Overall, these data highlight that perturbations in Mn homeostasis may alter multiple cellular processes, including iron (Fe) uptake. Intracellular cross talk between Mn and Fe homeostasis is not unprecedented. These two metal ions share many similar properties, and it was previously proposed that the Mn/Fe ratio may serve as an effective reporter for microbial resistance to host defenses during pathogenesis. The discoveries presented in the article by Rosch et al. support further research pursuits in the development of alternate metal-based antimicrobial therapeutics as a means to strategically treat infectious diseases.

The article by Rosch et al. was published during my Ph.D. research and has since impacted my thinking and research direction toward investigating the molecular underpinnings of why and how bacteria maintain optimal intracellular Mn levels. Similarly to other transition metals, intracellular Mn in bacteria is buffered by the absolute binding affinities of the metallobiomolecules that regulate the expression of the downstream genes which often encode metal efflux transporters like MntE. These regulatory metallobiomolecules sense bioavailable “free” Mn, which is a weakly competitive metal ion compared to other transition metals. Although collaborative efforts between microbiologists, structural biologists, and biophysical chemists have provided insight into the fundamental parameters that influence intermetal competition and mismetallation of these metallobiomolecules, relatively little information is available regarding the molecular mechanisms of how intracellular Mn is trafficked to target metalloenzymes in cells and why cells must avoid cellular Mn toxicity. Transport mutants incapable of Mn efflux permit researchers like myself to perturb Mn homeostasis and examine its cellular effects. Although my research is focused on the single metal ion Mn, I am mindful that Mn is part of a bigger metal milieu. It is apparent that intermetal competition or cross talk occurs between Mn and several other metal homeostasis pathways, including Fe, Zn, and Cu. This competition becomes substantially more complex at the host-pathogen interface, in which bacteria respond differently to distinct host responses. Continued collaborative efforts and interdisciplinary approaches are necessary to advance our understanding of metal cross talk within and among organisms. I foresee that transcriptomic profiles, such as those presented in the article by Rosch et al., and evolving proteomic approaches will play a big role in this area in the future.
